# Human Plasmacytoid and Monocyte-Derived Dendritic Cells Display Distinct Metabolic Profile Upon RIG-I Activation

**DOI:** 10.3389/fimmu.2018.03070

**Published:** 2018-12-21

**Authors:** Tünde Fekete, Mate I. Sütö, Dora Bencze, Anett Mázló, Attila Szabo, Tamas Biro, Attila Bacsi, Kitti Pazmandi

**Affiliations:** ^1^Department of Immunology, Faculty of Medicine, University of Debrecen, Debrecen, Hungary; ^2^MTA-DE Cell Biology and Signaling Research Group, University of Debrecen, Debrecen, Hungary

**Keywords:** plasmacytoid dendritic cell, dendritic cell, metabolic reprogramming, glycolysis, RIG-I, TLR, type I interferon, antiviral response

## Abstract

Recent advances reveal that metabolic reprogramming is required for adequate antiviral responses of dendritic cells (DCs) that possess the capacity to initiate innate and adaptive immune responses. Several reports indicate that Toll-like receptor (TLR) stimulation of DCs is accompanied by a rapid induction of glycolysis; however, the metabolic requirements of retinoic-acid inducible gene I (RIG-I)-like receptor (RLR) activation have not defined either in conventional DCs (cDCs) or in plasmacytoid DCs (pDCs) that are the major producers of type I interferons (IFN) upon viral infections. To sense viruses and trigger an early type I IFN response, pDCs rely on endosomal TLRs, whereas cDCs employ cytosolic RIG-I, which is constitutively present in their cytoplasm. We previously found that RIG-I is upregulated in pDCs upon endosomal TLR activation and contributes to the late phase of type I IFN responses. Here we report that TLR9-driven activation of human pDCs leads to a metabolic transition to glycolysis supporting the production of type I IFNs, whereas RIG-I-mediated antiviral responses of pDCs do not require glycolysis and rather rely on oxidative phosphorylation (OXPHOS) activity. In particular, TLR9-activated pDCs show increased extracellular acidification rate (ECAR), lactate production, and upregulation of key glycolytic genes indicating an elevation in glycolytic flux. Furthermore, administration of 2-deoxy-D-glucose (2-DG), an inhibitor of glycolysis, significantly impairs the TLR9-induced secretion of type I IFNs by human pDCs. In contrast, RIG-I stimulation of pDCs does not result in any alterations of ECAR, and type I IFN production is not inhibited but rather promoted by 2-DG treatment. Moreover, pDCs activated via TLR9 but not RIG-I in the presence of 2-DG are impaired in their capacity to prime allogeneic naïve CD8^+^ T cell proliferation. Interestingly, human monocyte-derived DCs (moDC) triggered via RIG-I show a commitment to glycolysis to promote type I IFN production and T cell priming in contrast to pDCs. Our findings reveal for the first time, that pDCs display a unique metabolic profile; TLR9-driven but not RIG-I-mediated activation of pDCs requires glycolytic reprogramming. Nevertheless, the metabolic signature of RIG-I-stimulated moDCs is characterized by glycolysis suggesting that RIG-I-induced metabolic alterations are rather cell type-specific and not receptor-specific.

## Introduction

DCs as part of the innate immune system constitute the first line of defense against viral infections playing a crucial role in both the recognition of foreign nucleic acids and subsequent triggering of antiviral responses (1). The innate immune response to viral infections is initiated when germ line-encoded pattern recognition receptors (PRRs) recognize specific viral molecular patterns (2). Upon binding to viral components, the main viral sensors such as endosomal TLRs and cytosolic RLRs induce signaling cascades that stimulate the rapid expression of genes encoding antiviral products like type I IFNs (3).

Plasmacytoid DCs are a rare subtype of DCs that are specialized in producing large amounts of type I IFNs in response to viruses (4). Unlike cDCs, pDCs are resistant to most viral infections and require a direct physical contact with infected cells or an uptake of virus-derived components released by them to successfully mount an antiviral state (5, 6). Plasmacytoid DCs are known to rely mainly on the endosomal TLR7 and TLR9 receptors to detect viral nucleic acids, whereas cDCs preferentially use cytosolic RLRs to recognize replicating viral RNA intermediates (2, 7). Intriguingly, recent findings including ours suggest that besides the TLR-mediated sensing of viral nucleic acids, RLRs are also involved in virus-triggered pDCs activation (8–11). We have recently found that RIG-I, a cytoplasmic sensors of viral RNA, is absent from quiescent pDCs but can be greatly upregulated upon endosomal TLR stimulation (8). Further we have proposed a model where endosomal TLRs mediate the first wave of type I IFN production while RIG-I contributes to the late phase of type I IFN responses in pDCs (8).

A growing body of evidence indicates that the activation of DCs does not only trigger changes in the expression of genes associated with immune responses but also induce metabolic reprogramming, which is important to meet the energetic needs of DC activation [reviewed in (12)]. Interestingly, to ensure optimal environment for replication, viruses also modulate host cellular metabolism inducing specific host metabolic pathways by distinct mechanisms (13). Various families of viruses have shown to alter core cellular metabolic pathways: most viruses induce glycolysis, whereas others induce fatty acid synthesis as well as glutaminolysis (14). In addition it has been recognized that there is a crosstalk between the immune system and cellular metabolism; immune cells can shift their metabolism in response to distinct microenvironmental stimuli e.g., viral infections (14). Recent evidence indicates that activation of DCs and macrophages is accompanied by rapid induction of glycolysis that provides adequate energy for activation and cytokine production [reviewed in (15)]. Moreover, in cDCs a range of TLR agonists has been found to induce a metabolic switch from OXPHOS to glycolysis which supports fatty acid synthesis that is required for DC activation (16, 17). Regarding the metabolic signature of activated pDCs only few studies are available, that all focus on endosomal TLR-driven metabolic alterations of pDCs (18–20). In particular, human pDCs show enhanced glycolytic activity upon stimulation with TLR7 specific respiratory viruses such as Flu and RV-16 virus and the synthetic TLR7 agonist gardiquimod (20). In contrast, activation of mouse pDCs through endosomal TLR9 resulted in increased OXPHOS and fatty acid oxidation (18). Furthermore, the authors demonstrated that the metabolic transition regulated through an autocrine type I IFN signaling loop is also characterized by changes in lipid metabolism that partially depends on the nuclear receptor peroxisome proliferator-activated receptor alpha (PPARα) in murine pDCs (18). All these findings imply that the activation-induced metabolic reprogramming of DCs might depend on the origin and source of the cells, the type of receptors as well as the activation signals.

As far as we know there are no data in the literature concerning the metabolic adaptation of DCs in response to RLR stimulation. Hence the primary goal of the present study is to address the link between cellular metabolism and RLR-mediated signal transduction in human DCs. In particular, we sought to explore the metabolic signature of RIG-I-activated human pDCs. Furthermore, we aimed to compare the metabolic requirements of RIG-I stimulated human pDCs and moDCs displaying distinct viral sensing machinery and different cytosolic RIG-I expression profile.

## Materials and Methods

### Cell Line

The human plasmacytoid dendritic cell line GEN2.2 (21) (provided by Dr. Joel Plumas and Dr. Laurence Chaperot, Research and Development Laboratory, French Blood Bank Rhône-Alpes, Grenoble, France) was used in our experiments, which is deposited with the CNCM (French National Collection of Microorganism Cultures) under the number CNCMI-2938. GEN2.2 cells were grown on a layer of mitomycin C (Sigma-Aldrich, St. Louis, MO, USA)-treated murine MS5 feeder cells (Cat. No. ACC 441, Leibniz Institute DSMZ-German Collection of Microorganisms and Cell Cultures, Braunschweig, Germany) in RPMI 1,640 medium (Sigma-Aldrich) supplemented with 10% heat-inactivated FBS (Life Technologies Corporation, Carlsbad, CA, USA), 100 U/ml penicillin, 100 μg/ml streptomycin (both from Sigma-Aldrich) and 5% non-essential amino acids (Life Technologies Corporation). For experiments, the GEN2.2 cells were removed from the feeder layer and seeded on 24-well plates at a concentration of 5 × 10^5^ cells/500 μl in complete RPMI 1,640 medium (Sigma-Aldrich). Cell lines were grown and incubated at 37°C in 5% CO_2_, at humidified atmosphere.

### Isolation and Culturing of Primary Human Cells

Human heparinized leukocyte-enriched buffy coats were obtained from healthy blood donors drawn at the Regional Blood Center of Hungarian National Blood Transfusion Service (Debrecen, Hungary) in accordance with the written approval of the Director of the National Blood Transfusion Service and the Regional and Institutional Ethics Committee of the University of Debrecen, Faculty of Medicine (Debrecen, Hungary).

Peripheral blood mononuclear cells (PBMC) were separated from buffy coats by Ficoll-Paque Plus (Amersham Biosciences, Uppsala, Sweden) gradient centrifugation. Monocytes were purified from PBMCs by positive selection using magnetic cell separation with anti-CD14-conjugated microbeads (Miltenyi Biotec, Bergish Gladbach, Germany) according to the manufacturer's instructions. Freshly isolated cells were seeded in 24-well cell culture plates at a density of 1 × 10^6^ cells/ml in RPMI 1,640 medium (Sigma-Aldrich) supplemented with 10% heat-inactivated FBS, 2 mM L-glutamine, 100 U/ml penicillin, 100 μg/ml streptomycin (all from Sigma-Aldrich), 80 ng/ml GM-CSF (Gentaur Molecular Products, London, UK), and 50 ng/ml IL-4 (PeproTech, Brussels, Belgium) for 5 days. On day 2, the half of the culture media was replaced with fresh media and the same amounts of GM-CSF and IL-4 were added to the cell cultures. Cells were used for experiments on day 5, when cells display immature DC phenotype (DC-SIGN/CD209^+^, CD14^−^, CD1a^+^).

Primary human pDCs were isolated from PBMCs by positive selection using the human CD304 (BDCA-4/Neuropilin-1) MicroBead Kit (Miltenyi Biotec) according to the manufacturer's instructions, then cultured in 96-well plates at a density of 1 × 10^5^ cells/200 μl in RPMI 1,640 medium (Sigma-Aldrich) supplemented with 10% heat-inactivated FBS (Life Technologies Corporation), 2 mM L-glutamine, 100 U/ml penicillin, 100 μg/ml streptomycin (all from Sigma-Aldrich), and 50 ng/ml recombinant human IL-3 (PeproTech).

Allogeneic naïve CD8^+^ T cells were isolated from PBMC using the human naïve CD8^+^ T cell isolation kit (Miltenyi Biotec) according to the manufacturer's instructions and were used for co-culture experiments as described below.

### Cell Stimulation

For TLR activation GEN2.2 cells and primary human pDCs were treated with TLR9 agonist CpG-A (ODN 2216, 1 μM; Hycult Biotech, Uden, The Netherlands) for 12 h. To induce RIG-I expression GEN2.2 cells and primary human pDCs were pre-treated with low dose of CpG-A (0.25 μM) for 16 h. Thereafter the cells were washed, re-seeded in fresh, complete RPMI 1,640 medium and stimulated with 5′ppp-dsRNA (InvivoGen, San Diego, CA, USA), a specific agonist of RIG-I in complex with the transfection reagent LyoVec^TM^ (InvivoGen), according to the manufacturer's recommendations. Briefly, 25 μl of the 5′ppp-dsRNA-LyoVec^TM^ complex containing 1 μg/ml working concentration of the RIG-I ligand was added to the cells for the indicated time periods in all experiments. For moDCs, on day 5 of the differentiation half of the culture medium was removed, replaced by fresh medium then cells were exposed to 5′ppp-dsRNA-LyoVec^TM^ complexes for 12 h. In parallel experiments cells were treated with indicated concentrations of the glycolysis inhibitor 2-deoxy-D-glucose (2-DG, Sigma-Aldrich) or OXPHOS inhibitor carbonylcyanide m-chlorophenylhydrazone (CCCP, Sigma-Aldrich).

### Determination of Cell Viability

Cell viability was assessed by 7-aminoactinomycin D (7-AAD; 10 μg/ml; Sigma–Aldrich) staining for 15 min immediately before flow cytometric analysis. Fluorescence intensities were measured with FACS Calibur cytometer (Becton Dickinson, Franklin Lakes, NJ, USA) and data were analyzed with FlowJo software (TreeStar, Ashland, OR, USA).

### Quantitative Real-Time PCR

Total RNA was isolated from 5 × 10^5^ cells using Tri reagent (Molecular Research Center, Inc., Cincinnati, OH, USA). One microgram of total RNA was treated with DNase I (Thermo Fisher Scientific, Waltham, MA, USA) to exclude amplification of genomic DNA then reverse transcribed into cDNA using the High Capacity cDNA RT Kit of Applied Biosystems (Foster City, CA, USA). Gene expression assays were purchased from Thermo Fisher Scientific for *IFNB, hexokinase 2 (HK2), lactate dehydrogenase A (LDHA), hypoxia-inducible factor 1-alpha (HIF1A)*, and from Integrated DNA Technologies (Coralville, IA, USA) for *IFNA1* and *PPIA* (cyclophilin A). Quantitative PCR was performed using the ABI StepOne Real-Time PCR System (Applied Biosystems) and cycle threshold values were determined using the StepOne v2.1 Software (Applied Biosystems). The relative amount of mRNA (2^−Δ^CT) was obtained by normalizing to the *PPIA* (Integrated DNA Technologies) housekeeping gene in each experiment.

### Assessment of Cytokine Levels and Lactic Acid From the Supernatants of Cell Cultures

Cell culture supernatants were collected at the indicated time points and IFN-α and IFN-β levels were measured by the VeriKine^TM^ Human Interferon Alpha and Beta ELISA kits, respectively, (PBL Interferon Sources, Piscataway, NJ, USA) according to the manufacturer's instructions. Lactate production of the cells was detected using the Glycolysis Cell-Based Assay Kit (Cayman Chemical, Ann Arbor, Michigan, USA) according to the manufacturer's instructions. Absorbance measurements were carried out by a Synergy HT microplate reader (Bio-Tek Instruments, Winooski, VT, USA) at 450 nm for cytokine detection and at 490 nm for lactate assay.

### Real-Time Extracellular Flux Analysis

Human pDCs and moDCs were harvested, washed and resuspended in Agilent Seahorse XF Base Medium (pH 7.4; Agilent Technologies, Santa Clara, CA, USA) supplemented with 10 mM glucose, 2 mmol/L glutamine and 1% FBS and seeded onto Cell-Tak (Corning Inc., NY, USA)-coated Seahorse XF96 Cell Culture Microplates (Agilent Technologies) at a density of 1.5 × 10^5^ cells per well. Cells were incubated at 37°C in a CO_2_-free incubator for 1 h before the experiments. Extracellular acidification rate (ECAR) and oxygen consumption rate (OCR) were measured simultaneously in real-time with a Seahorse XF96^e^ Extracellular Flux Analyzer (EFA; Agilent Technologies). The compounds, CpG-A and 5′ppp-dsRNA were added immediately before EFA measurements.

### Detection of Mitochondrial Reactive Oxygen Species (mtROS)

Primary pDCs and moDCs were loaded with 5 μM MitoSox^TM^ Red mitochondrial superoxide indicator (Life Technologies Corporation) and incubated for 10 min at 37°C protected from light. Then cells were washed gently three times with warm PBS buffer (Sigma-Aldrich) to remove the excess fluorescent dye and plated in 96-well black polystyrene plate at a density of 2 × 10^5^ cells/200 μl in RPMI 1,640 medium (Sigma-Aldrich). Cells were then left untreated or stimulated with 5′ppp-dsRNA as described above. Fluorescence intensity of MitoSox™ Red was recorded at 580 nm by a Synergy HT microplate reader (Bio-Tek Instruments).

### Western Blotting

For western blotting 5 × 10^5^ cells were lysed in Laemmli buffer and then the protein extracts were resolved by SDS-PAGE using 10% polyacrylamide gel and electro-transferred to nitrocellulose membranes (Bio-Rad Laboratories GmbH, Munich, Germany). Non-specific binding sites were blocked with 5% non-fat dry milk diluted in TBS Tween buffer (50 mM Tris, 0.5 M NaCl, 0.05% Tween-20, pH 7.4). Membranes were probed with the anti-RIG-I (Cat. No. 4520, Cell Signaling, Danvers, MA, USA) and anti-beta-actin (Cat. No. sc-47778, Santa Cruz Biotechnology) primary antibodies. The bound primary antibodies were labeled with anti-mouse or anti-rabbit horseradish peroxidase-conjugated secondary antibodies (GE Healthcare, Little Chalfont, Buckinghamshire, UK) at a dilution of 1:5,000 and 1:10,000, respectively and were visualized by the ECL system using SuperSignal West Pico chemiluminescent substrate (Thermo Scientific, Rockford, IL, USA) and X-ray film exposure. Densitometric analysis of immunoreactive bands was performed using Image Studio Lite Software version 5.2 (LI-COR Biosciences, Lincoln, Nebraska USA).

### T Cell Proliferation Assay

Prior to co-culture with allogeneic naïve CD8^+^ T cells primary human pDCs were stimulated with CpG-A (1 μM) for TLR9 activation in the presence or absence of 2-DG for 6 h. In parallel experiments, primary pDCs were pre-treated with CpG-A (0.25 μM) for 16 h to induce RIG-I expression as described above then following thorough washing steps stimulated with the specific RIG-I ligand 5′ppp-dsRNA with or without 2-DG for 6 h. Immature moDCs were plated and stimulated with 5′ppp-dsRNA for 6 h in the presence or absence of glycolysis inhibitor. Following incubation activated DCs were washed twice with cell culture medium then co-cultured in 96-well U-bottom plate with allogeneic naïve CD8^+^ T cells, which were previously labeled with 0.5 μM of carboxyfluorescein succinimidyl ester (CFSE; Invitrogen, Carlsbad, CA, USA), for 5 days in the presence of 1 μg/ml anti-human CD3 monoclonal antibody (BD Pharmingen) at a ratio of 1:10 (DC-T cell). After co-cultivation, fluorescence intensities of CFSE dye were detected in the FL1 (530 ± 15 nm) channel on a BD FACS Calibur flow cytometer (Becton Dickinson) and data were analyzed by FlowJo software (Treestar).

### Statistical Analysis

Multiple comparisons were performed using ANOVA, followed by Bonferroni *post-hoc* test whereas two groups were compared with Student's unpaired *t*-test. Data analyses were performed using GraphPad Prism v.6 software (GraphPad Software Inc., La Jolla, CA, USA). Differences were considered to be statistically significant at *p* < 0.05.

## Results

### Plasmacytoid DCs Display Distinct RIG-I Expression Profile Compared to moDCs

**Figure 1:**
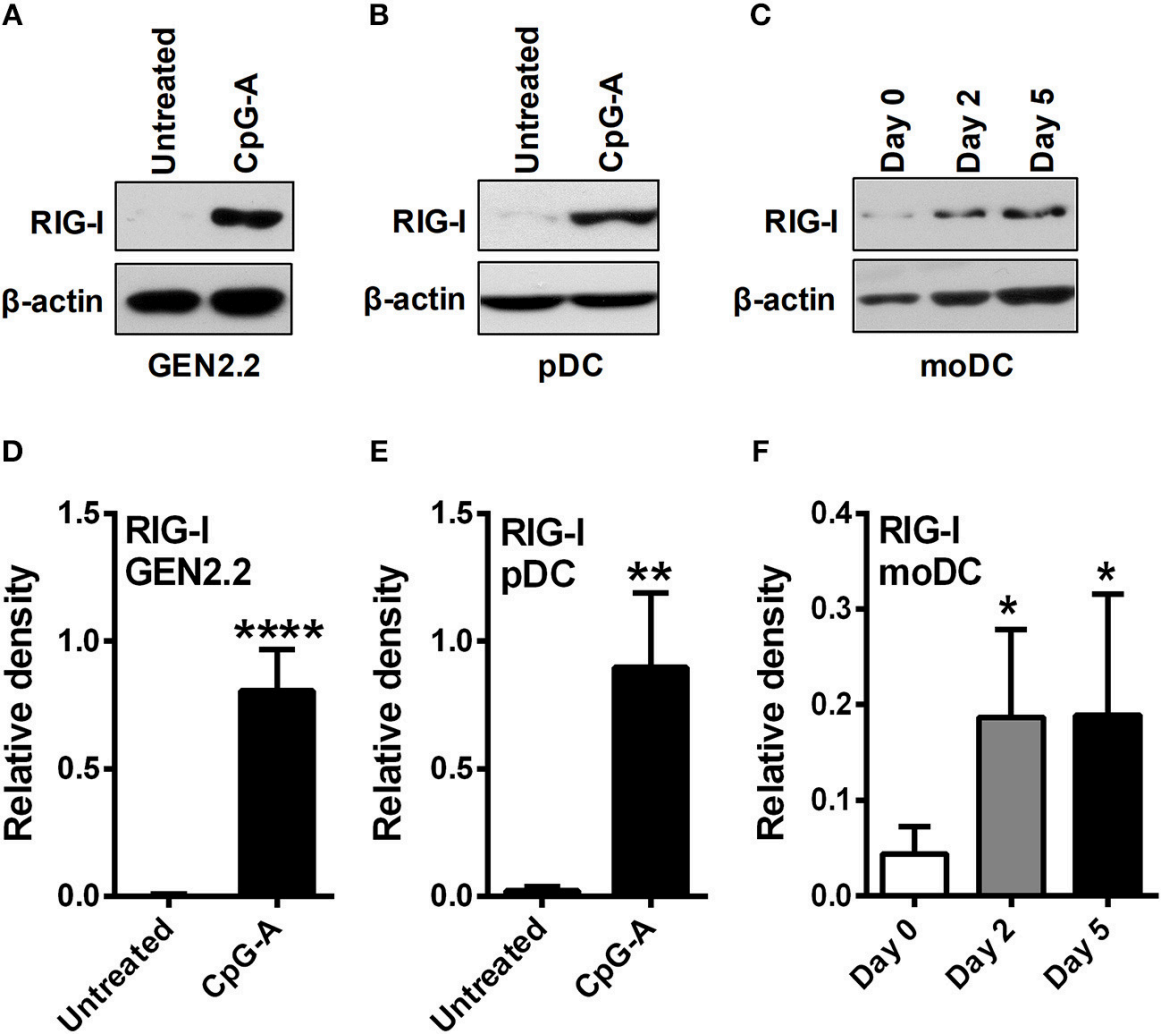
Plasmacytoid DCs display distinct RIG-I expression profile compared to moDCs. GEN2.2 cells **(A,D)** and primary human pDCs **(B,E)** were treated with TLR9 agonist CpG-A (0.25 μM) for 16 h then the protein level of RIG-I was determined by western blotting. **(C**,**F)** Freshly isolated monocytes were seeded in 24-well plates and differentiated as described in the Materials and Methods. The protein level of RIG-I was measured by western blotting. Representative blots are shown in **(A–C)**. Data are shown as mean ± SD of at least three independent measurements in **(D–F)**. *p < 0.05, **p < 0.01, ****p < 0.0001.

Due to the limited number of pDCs in human peripheral blood we performed most of our experiments on the human pDC cell line GEN2.2 that shares similar phenotypic and functional properties with primary human pDCs (22, 23). Furthermore, our main findings have been validated in primary human pDCs isolated from peripheral blood of healthy volunteers. Besides we have used moDCs generated from human peripheral blood monocytes *in vitro* as it serves as an ideal model for studying DC functionality (24). First we investigated the expression profile of RIG-I in these DC subtypes. Previously we have published that GEN2.2 cells require a pre-treatment with TLR9 agonist CpG-A to express the cytosolic RIG-I receptor (25, 26) as also shown in Figures 1A,D of the present study. Similarly to GEN2.2 cells RIG-I is also absent from resting primary pDCs (8, 26) but can be significantly upregulated upon exposure to CpG-A (Figures 1B,E) showing a unique RIG-I expression profile in pDCs. On the contrary, RIG-I was gradually upregulated during moDC differentiation, and was constantly present in 5 day immature moDCs (Figures 1C,F) (26). Therefore, these two DC subtypes provide potential models to study the RIG-I-induced metabolic changes in cell types with different RIG-I expression profile, namely in moDCs that constitutively express RIG-I and in pDCs with inducible RIG-I expression.

### Inhibition of Glycolysis Influences the Viability and RIG-I Expression of GEN2.2 Cells

**Figure 2:**
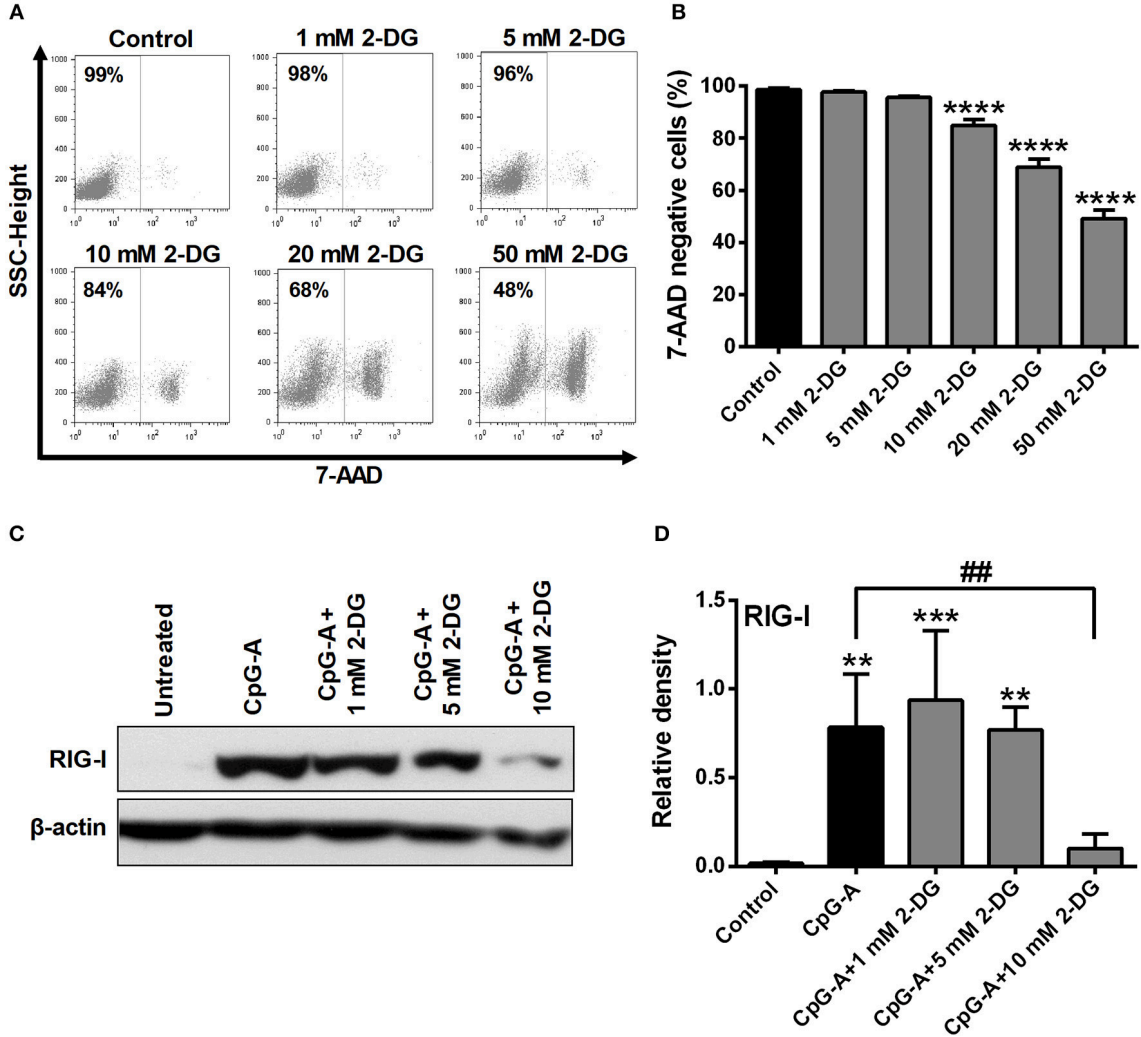
Inhibition of glycolysis influences the viability and RIG-I expression of GEN2.2 cells in a concentration-dependent manner. **(A,B)** GEN2.2 cells were treated with increasing concentration of 2-deoxy-D-glucose (2-DG; 1–50 mM) then cell viability was analyzed by flow cytometry. **(C,D)** GEN2.2 cells were left untreated, treated with TLR9 ligand CpG-A (0.25 μM) alone or in combination with increasing concentrations of 2-DG (1–10 mM) for 16 h then the protein level of RIG-I was measured by western blot. **(A)** Representative dot plots are shown, where numbers indicate the percentage of 7-aminoactinomycin D (7-AAD) negative cells. **(B)** Bar graphs show the mean ± SD of four independent experiments. **(C)** Representative blot is shown. **(D)** Bar graphs represent the mean ± SD of four individual experiments. **p < 0.01, ***p < 0.001 ****p < 0.0001 vs. control; ^##^*p* < 0.01.

Growing data support the idea that activation of DCs with various TLR agonists is coupled with a metabolic transition (17, 20). To investigate the role of glycolysis in pDC activation, cells were treated with the potent glycolysis inhibitor 2-deoxy-D-glucose (2-DG). First we titrated 2-DG to determine the optimal concentration that would be tolerated by GEN2.2 cells. Our results show that low doses of 2-DG (1–5 mM) do not or only slightly (10 mM) affected cell viability, whereas higher doses (20–50 mM) were not tolerated by GEN2.2 cells (Figures 2A,B). Based on our dose-response curve we have decided to use 1, 5, and 10 mM of 2-DG to our further experiments which concentrations of 2-DG did not markedly increase the ratio of 7-aminoactinomycin D (7-AAD) positive cells in the cell cultures.

Investigating the impact of glycolysis blockade on RIG-I expression we found that 1 and 5 mM concentrations of 2-DG did not influence the CpG-A induced expression of RIG-I in GEN2.2 cells, whereas 10 mM of 2-DG decreased its protein levels significantly (Figures 2C,D). These results indicate that the RIG-I expression can be controlled by glycolysis in pDCs.

### TLR but Not RLR Stimulation Requires a Shift Toward Glycolysis to Induce a Robust Type I IFN Production in GEN2.2 Cells

**Figure 3:**
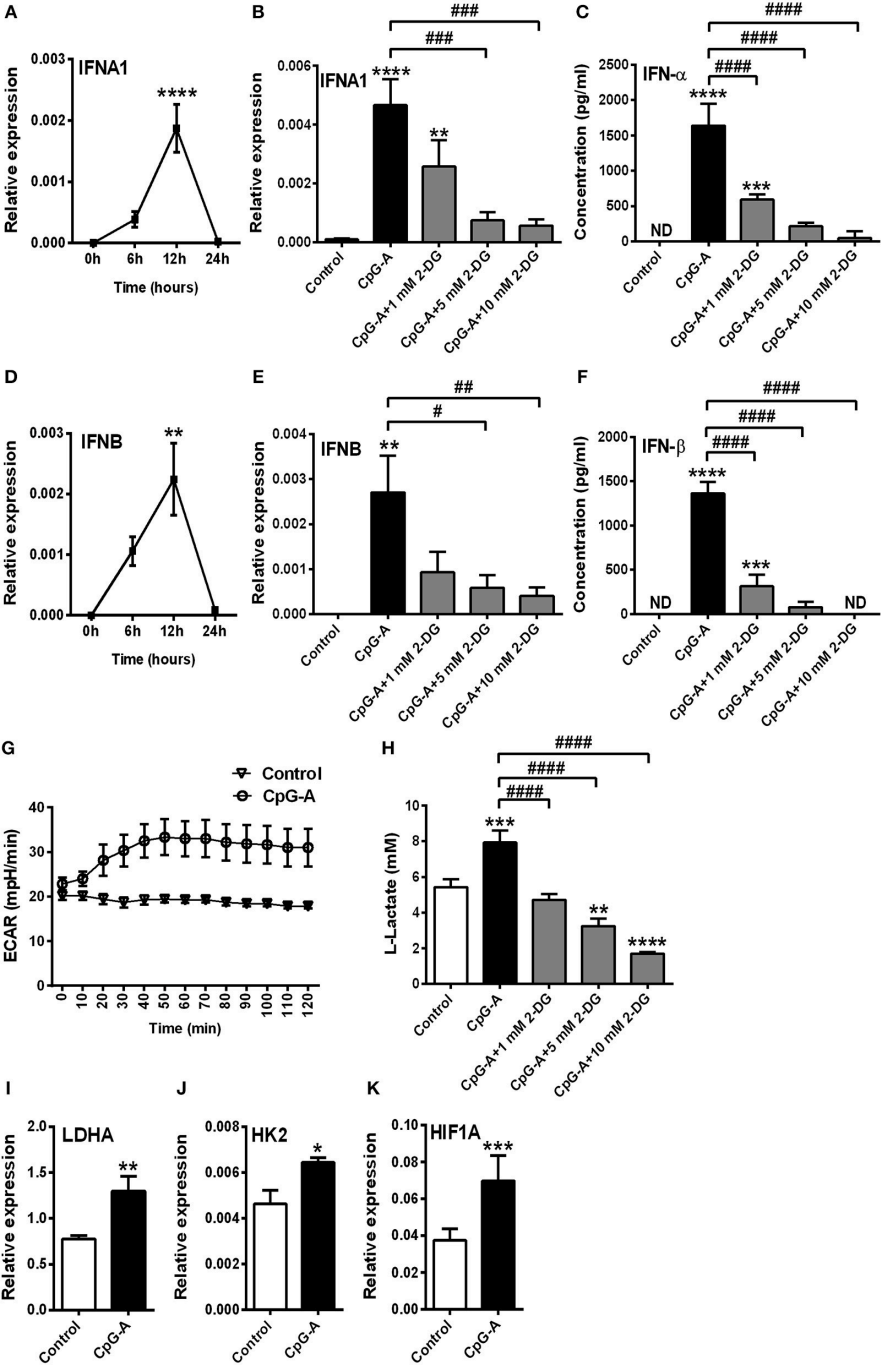
A shift to glycolysis is essential to the CpG-A-induced production of type I IFNs in GEN2.2 cells. **(A,D)** GEN2.2 cells were treated with 1 μM of CpG-A, and the expression of *IFNA1* and *IFNB* was measured in a time-dependent manner at the mRNA level by Q-PCR. **(B,C,E,F)** GEN2.2 cells were left untreated, treated with 1 μM of CpG-A alone or in combination with increasing concentrations of 2-deoxy-D-glucose (2-DG; 1–10 mM) for 12 h. The IFN-α and IFN-β expression was assessed by real-time PCR at the mRNA level **(B,E)** and by ELISA at the protein level **(C,F)**. **(G)** Following activation with CpG-A real-time ECAR of GEN2.2 cells was determined by EFA. The results of a representative experiment are shown. **(H)** Lactate concentrations were measured from the supernatants at 12 h. The expression of *LDHA* **(I)**, *HK2* **(J)**, and *HIF1A* **(K)** was assessed at the mRNA level by real-time PCR. Figures represent the mean ± SD of 4–6 independent experiments. *p < 0.05, **p < 0.01, ***p < 0.001 ****p < 0.0001 vs. control; ^#^*p* < 0.05, ^##^*p* < 0.01, ^###^*p* < 0.001, ^####^*p* < 0.0001, ND, not determined.

Previously we have described that following recognition of viral nucleic acids the type I IFN production of pDCs occurs in two waves (8). Endosomal TLRs mediate early type I IFN production; whereas cytosolic RLRs induced by TLR stimulation substantially contribute to the late phase of IFN responses. We sought to test the role of glycolysis in both the first and second phase of type I IFN responses in human pDCs. First we used the TLR9 ligand CpG-A (1 μM) to induce early type I IFN production in GEN2.2 cells. Time-dependent analysis of *IFNA1* and *IFNB* expression shows a peak at 12 h following CpG-A stimulation (Figures 3A,D); therefore, we have studied the effect of glycolysis at this time point. Inhibition of glycolysis by 2-DG interrupted the CpG-A induced expression of IFN-α and IFN-β in a dose-dependent manner both at the mRNA (Figures 3B,E) and protein level (Figures 3C,F) indicating a critical role for glycolysis in these processes. The glycolysis inhibitor applied alone did not induce type I IFN production at any of the used concentrations (data not shown); therefore we excluded those treatment conditions from subsequent experiments. Next we measured real-time ECAR, an indicator of glycolysis, and found that GEN2.2 cells increased their ECAR levels following CpG-A administration (Figure 3G). Consistent with this, the elevated lactate production of CpG-A-stimulated cells indicates an increase in glycolytic activity that could be inhibited by administration of 2-DG (Figure 3H). We have also analyzed the changes in the expression of key glycolytic genes in CpG-A-exposed GEN2.2 cells at the mRNA level and we found that *LDHA, HK2*, and *HIF1A* are significantly upregulated upon exposure to CpG-A (Figures 3I–K). All these results indicate that endosomal TLR9 stimulation requires glycolysis to induce type I IFN secretion in GEN2.2 cells.

**Figure 4:**
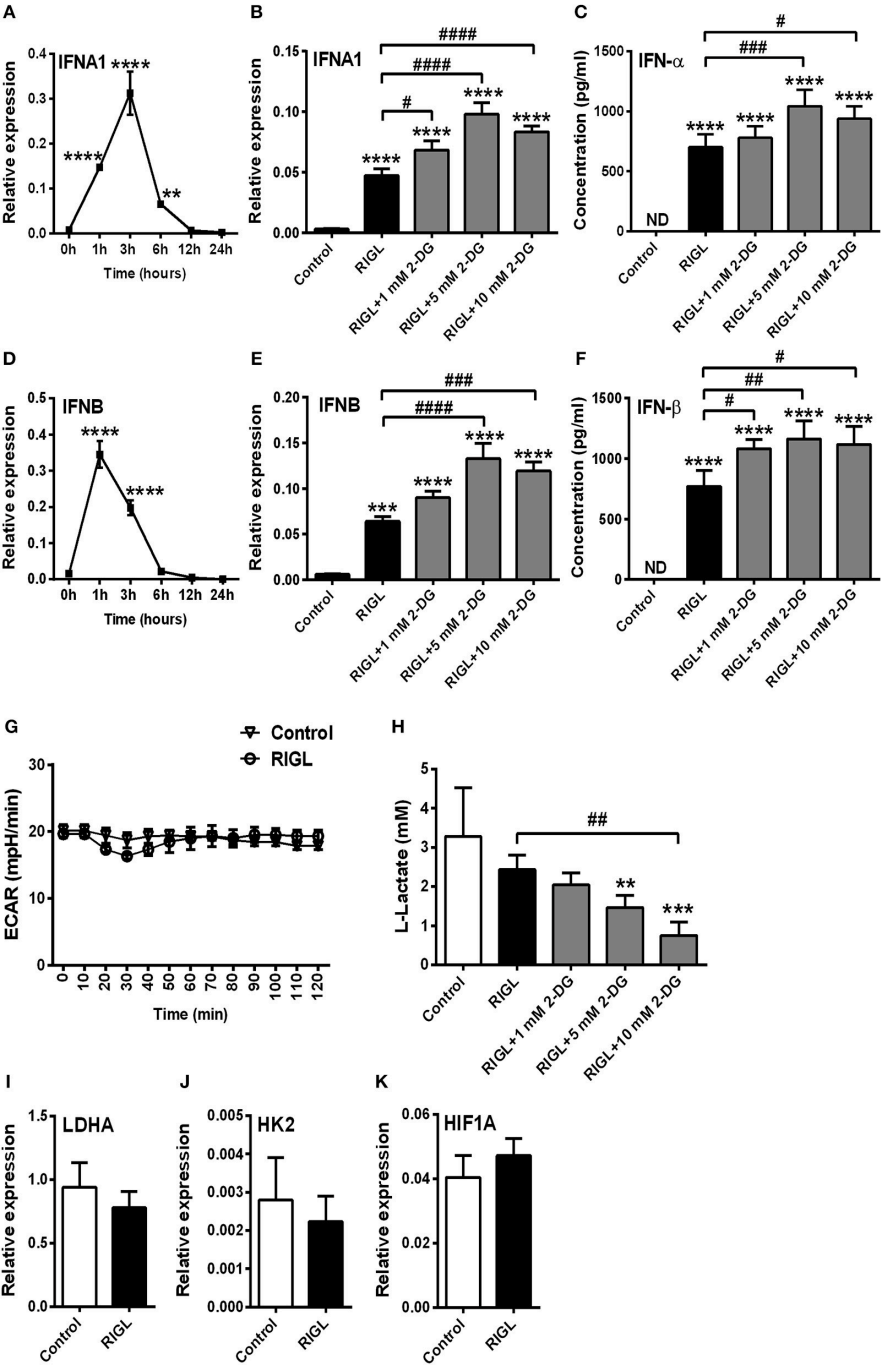
Glycolysis is not required to the RIG-I-mediated type I IFN production in GEN2.2 cells. **(A,D)** GEN2.2 cells were pre-treated with 0.25 μM of CpG-A for 16 h then following thorough washing steps stimulated with the RIG-I agonist 5′ppp-dsRNA (RIGL, 1 μg/ml) in a time-dependent manner. The mRNA level of *IFNA1* and *IFNB* was measured by Q-PCR. **(B,C,E,F)** After pre-treatment with low dose of CpG-A, GEN2.2 cells were exposed to 5′ppp-dsRNA in the absence or presence of the indicated concentrations of 2-deoxy-D-glucose (2-DG; 1–10 mM). The IFN-α and IFN-β expression was assessed by real-time PCR at the mRNA level **(B,E)** and by ELISA at the protein level **(C,F)**. **(G)** Following activation with 5′ppp-dsRNA real-time ECAR of GEN2.2 cells was determined by EFA. The results of a representative experiment are shown. **(H)** Lactate levels were measured from the supernatants of the cell cultures. The expression of *LDHA* **(I)**, *HK2* **(J)**, and *HIF1A* **(K)** was assessed at the mRNA level by real-time PCR. Data represent the mean ± SD of at least three independent experiments. **p < 0.01, ***p < 0.001, ****p < 0.0001 vs. control; ^#^*p* < 0.05, ^##^*p* < 0.01, ^###^*p* < 0.001, ^####^*p* < 0.0001, ND, not determined.

Next we asked whether pDC activation in response to RIG-I stimulation is also accompanied by a shift toward glycolysis. Therefore, GEN2.2 cells were pre-treated with low dose of CpG-A (0.25 μM) for 16 h to induce the cytosolic expression of RIG-I then following thorough washing steps stimulated with the specific RIG-I agonist 5′ppp-dsRNA. We have previously developed and applied this method successfully to study RLR responses in pDCs since a pre-treatment with low dose of CpG-A does not result in cell exhaustion (8, 25). The activation of pDCs with a RIG-I ligand causes a more rapid type I IFN response than the activation with a TLR9 ligand. *IFNA1* and *IFNB* mRNA expression peaks at 1–3 h after RIG-I stimulation thus we have studied the impact of glycolysis inhibition at 3 h (Figures 4A,D). Interestingly, disruption of glycolysis by 2-DG significantly increased the 5′ppp-dsRNA-induced expression of IFN-α and IFN-β both at the mRNA and protein level (Figures 4B,C,E,F). Further we found that pDC activation in response to 5′ppp-dsRNA was not accompanied by an increase in ECAR (Figure 4G) in contrast to TLR9 activation. These results were supported by the findings that RIG-I stimulation did not give rise to elevated lactate production (Figure 4H) or upregulation of glycolysis-associated genes (Figures 4I–K). All these results imply that RIG-I-mediated type I IFN responses do not depend on glycolysis and instead use other metabolic pathways to ensure energy for the production of late type I IFN secretion.

**Figure 5:**
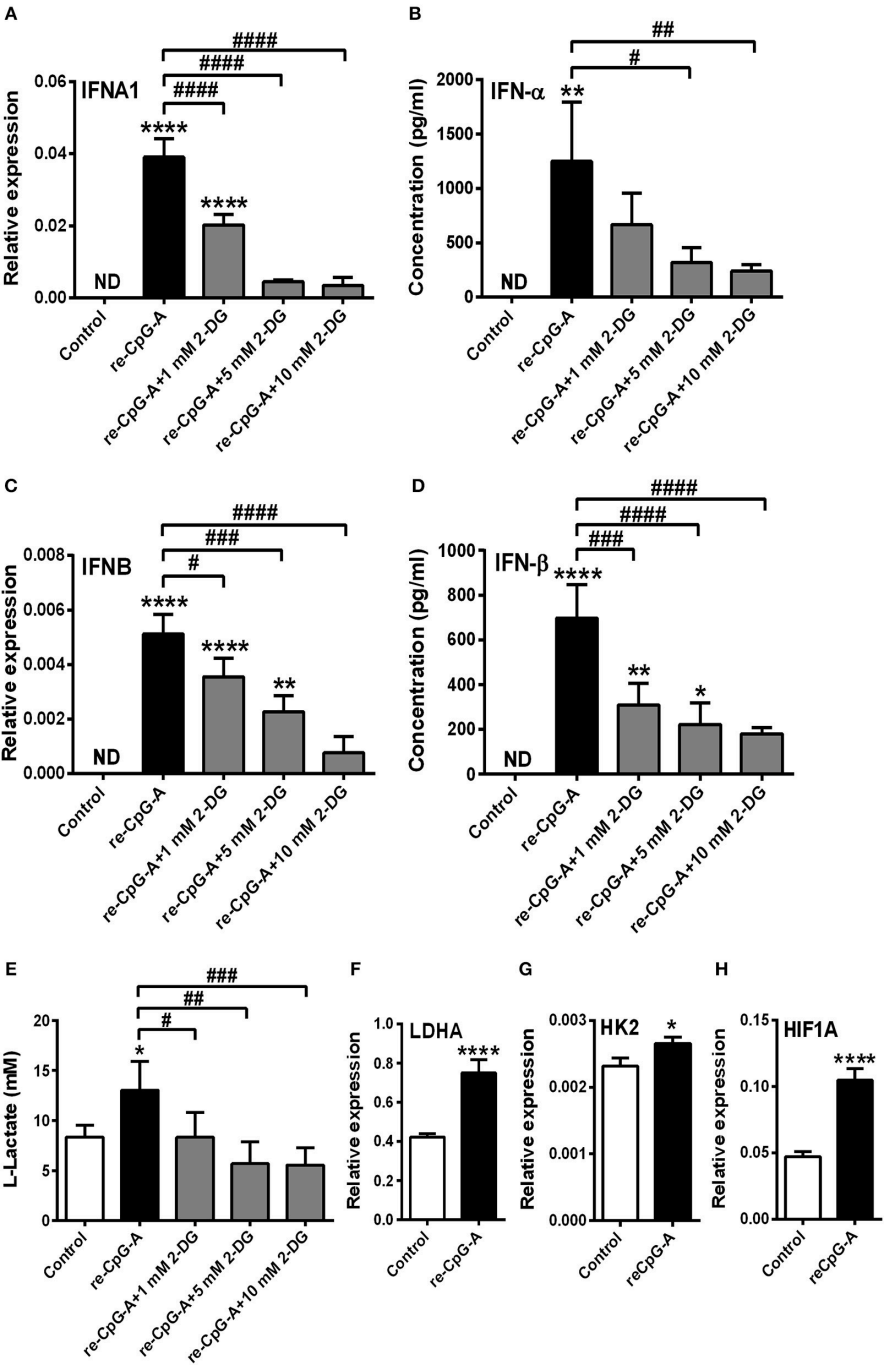
The type I IFN production of GEN2.2 cells induced by a second exposure to CpG-A also depends on glycolytic metabolism. GEN2.2 cells were pre-treated with 0.25 μM of CpG-A for 16 h then following thorough washing steps re-stimulated with 1 μM CpG-A (re-CpG-A) in the absence or presence of 2-deoxy-D-glucose (2-DG; 1–10 mM). The *IFNA1* and *IFNB* mRNA expression level was assessed by real-time PCR **(A,C)** and the IFN-α and IFN-β protein level was measured by ELISA **(B,D)** at 12 h. **(E)** Lactate concentrations were determined from the supernatants of the cells at 12 h. The expression of *LDHA* **(F)**, *HK2* **(G)**, and *HIF1A* **(H)** was assessed at the mRNA level by real-time PCR. **(A–H)** Bar graphs represent the mean ± SD of four independent experiments. *p < 0.05, **p < 0.01, ****p < 0.0001 vs. control; ^#^*p* < 0.05, ^##^*p* < 0.01, ^###^*p* < 0.001, ^####^*p* < 0.0001, ND, not determined.

In parallel experiments after the pre-treatment with low dose of CpG-A (0.25 μM) we used high dose of CpG-A (1 μM) instead of RIG-I ligand as a second stimulus to exclude the possibility that a preceding activation modifies the metabolic requirements to a subsequent stimulus. Here we observed that re-stimulation with CpG-A also requires glycolysis to induce IFN-α production in GEN2.2 cells (Figure 5). Particularly, blockade of glycolysis by 2-DG dampened IFN-α and IFN-β expression both at the mRNA (Figures 5A,C) and protein level following re-stimulation with CpG-A (Figures 5B,D). Furthermore, a second stimulus with CpG-A increased lactate production, which was inhibited by 2-DG administration (Figure 5E), and upregulated *LDHA, HK2*, and *HIF1A* mRNA levels significantly (Figures 5F–H). All these results suggest that enhanced glycolysis dominates both early and late TLR9 responses, whereas RIG-I-mediated signaling does not rely on it in GEN2.2 cells.

### TLR but Not RLR Stimulation Enhances Glycolysis to Induce the Production of Type I IFNs Even in Primary Human pDCs

**Figure 6:**
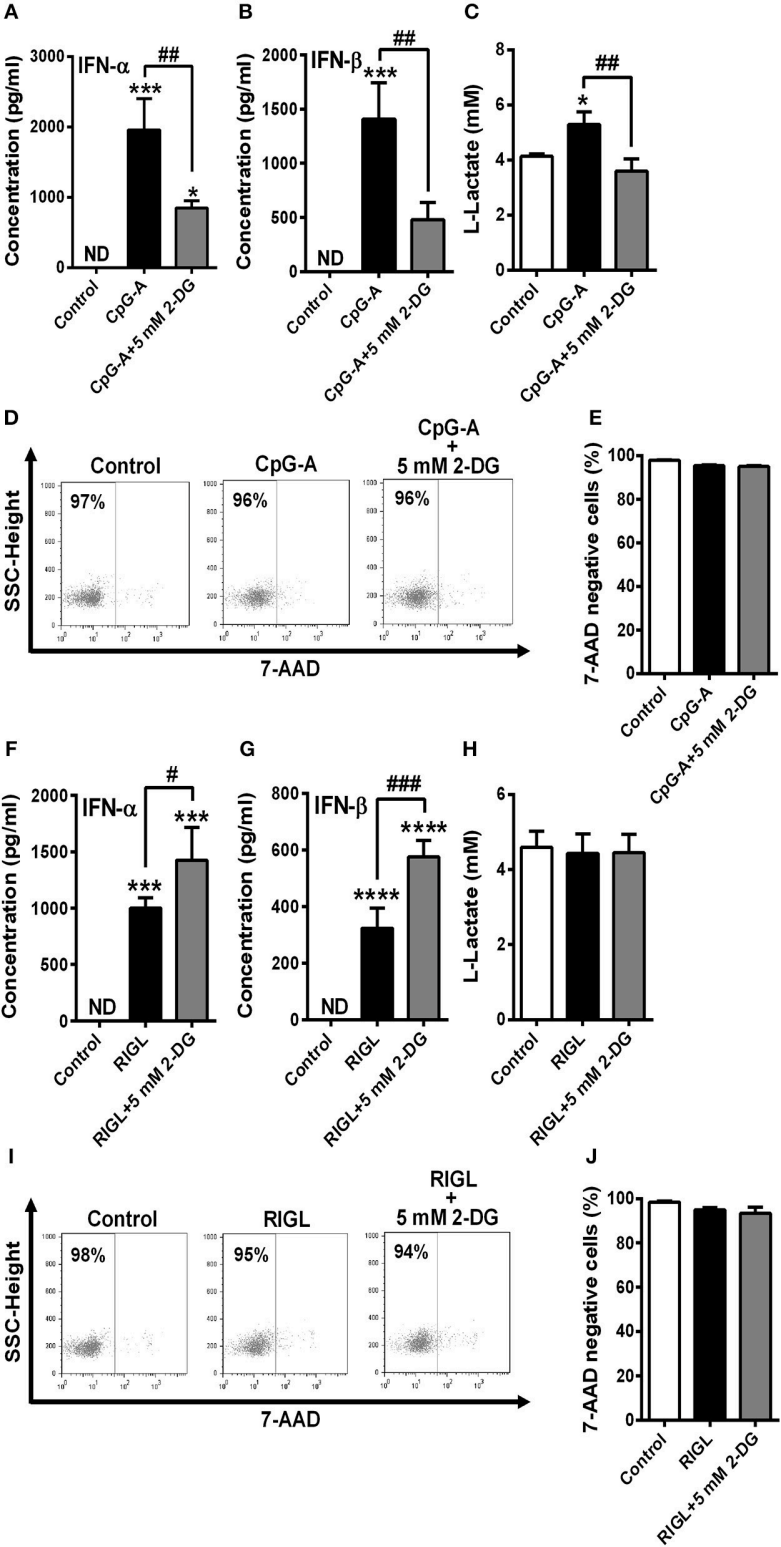
TLR9 but not RIG-I activation requires glycolysis to induce the production of type I IFNs in primary human pDCs. Freshly isolated primary human pDCs were stimulated with 1 μM of CpG-A in the absence or presence of 2-deoxy-D-glucose (2-DG; 5 mM) then IFN-α and IFN-β protein levels **(A,B)** and lactate concentrations **(C)** were measured from the supernatants of the cells at 12 h. In parallel experiments cells were pre-treated with low dose of CpG-A for 16 h then following thorough washing steps stimulated with 5′ppp-dsRNA (RIGL, 1 μg/ml) alone or in combination with 5 mM of 2-DG or left untreated. IFN-α and IFN-β protein levels **(F,G)** and lactate concentrations **(H)** were measured from the supernatants at 6 h. **(D,E,I,J)** Cell viability was measured by 7-aminoactinomycin D (7-AAD) staining using flow cytometry. **(D,I)** Representative dot plots are shown where numbers indicate the percentage of 7-AAD negative cells. **(A–C,E,F–H,J)** Data represent the mean ± SD of three individual experiments. *p < 0.05, ***p < 0.001, ****p < 0.0001 vs. control; ^#^*p* < 0.05, ^##^*p* < 0.01, ^###^*p* < 0.001, ND, not determined.

To confirm our results we have also repeated our experiments with primary human pDCs. Due to the limited cell number only one concentration of 2-DG (5 mM) was tested that did not alter cell viability neither of GEN2.2 cells (Figures 2A,B) nor of primary pDCs (Figures 6D,E,I,J). Our results are in line with the data obtained by studies on the GEN2.2 cell line. The CpG-A-induced IFN-α and IFN-β production of primary pDCs was impaired in the presence of 2-DG (Figures 6A,B), whereas RIG-I-mediated IFN-α and IFN-β secretion was rather further increased in the presence of the glycolysis inhibitor (Figures 6F,G). Further we observed elevated lactate production in the supernatants of CpG-A stimulated cells that was reduced when cells were co-treated with 2-DG (Figure 6C). On the contrary, we did not observe any changes in the lactate levels of RIG-I stimulated pDCs (Figure 6H) indicating that RIG-I activation does not engage glycolysis and might use different metabolic pathways to serve macromolecule/protein synthesis.

In order to further investigate the metabolic signature of RIG-I stimulated GEN2.2 cells and primary pDCs we wanted to study the importance of OXPHOS in RIG-I signaling. Therefore, OXPHOS was uncoupled by the addition of potent OXPHOS inhibitor carbonyl cyanide m-chlorophenyl hydrazone (CCCP) (Supplementary Figure 1). First we determined the optimal concentrations (1 and 5 μM) of CCCP to treat GEN2.2 cells (Supplementary Figures 1A,B), then observed that RIG-I-induced IFN-α and IFN-β production of GEN2.2 cells was reduced in the presence of CCCP (Supplementary Figures 1C–F). Performing real-time measurements of OCR in pDCs we observed a minimal increase in OCR upon RIG-I stimulation (Supplementary Figure 1G). In primary human pDCs CCCP co-treatment also showed negative impact on the RIG-I induced IFN-α and IFN-β secretion (Supplementary Figures 2A,B) without influencing the viability of the cells (Supplementary Figures 2C,D). Furthermore, we detected elevated mtROS levels in GEN2.2 cells and primary pDCs after RIG-I activation (Supplementary Figures 1H, 2E).

All these observations support the hypothesis that in contrast with TLR9 activation, human pDCs activated via RIG-I do not require glycolysis and might favor OXPHOS to produce type I IFNs.

### Glycolysis Is Essential to the RIG-I-Induced Type I IFN Production of moDCs

**Figure 7:**
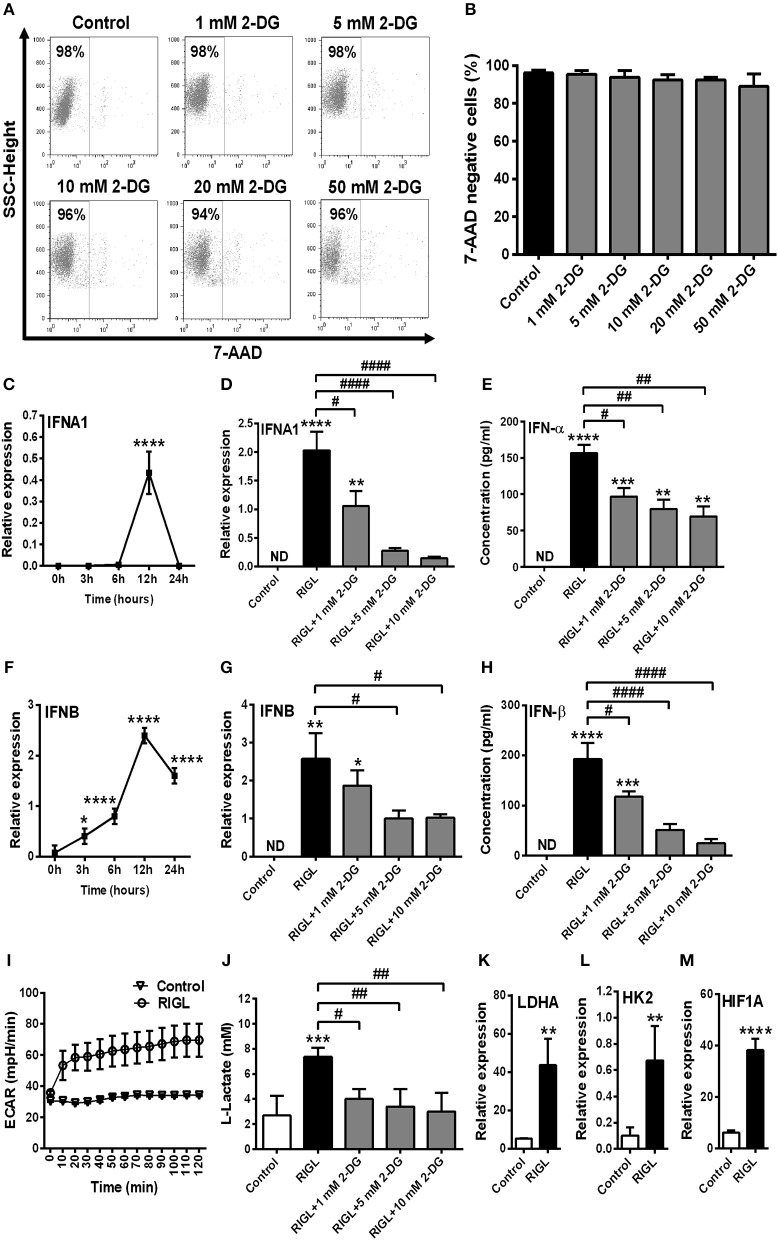
Glycolytic switch is required to the RIG-I-mediated type I IFN production in moDCs. **(A,B)** Immature moDCs were treated with increasing concentration of 2-deoxy-D-glucose (2-DG; 1–50 mM) then cell viability was analyzed by flow cytometry. **(C,F)** Immature moDCs were stimulated with the RIG-I agonist 5′ppp-dsRNA (RIGL, 1 μg/ml) in a time-dependent manner. Kinetics expression of *IFNA1* and *IFNB* mRNA was measured by Q-PCR. **(D,E,G,H)** In parallel experiments moDCs were treated with RIG-I ligand in the absence or in the presence of the indicated concentrations of 2-deoxy-D-glucose (2-DG; 1–10 mM) for 12 h. The IFN-α and IFN-β expression was assessed by real-time PCR at the mRNA level **(D,G)** and by ELISA at the protein level **(E,H)**. **(I)** Following activation with RIG-I agonist 5′ppp-dsRNA, real-time ECAR of moDCs was determined by EFA. The results of a representative experiment are shown. **(J)** Lactate concentrations were measured from the supernatants at 12 h. The expression of *LDHA* **(K)**, *HK2* **(L)**, and *HIF1A* **(M)** was assessed at the mRNA level by real-time PCR. **(A)** Representative dot plots are shown where numbers indicate the percentage of 7-aminoactinomycin D (7-AAD) negative cells. **(B–M)** Data represent the mean ± SD of 4 independent experiments. *p < 0.05, **p < 0.01, ***p < 0.001, ****p < 0.0001 vs. control; ^#^*p* < 0.05, ^##^*p* < 0.01, ^####^*p* < 0.0001, ND, not determined.

In order to test whether these results are specific to human pDCs we extended our studies on immature moDCs, which constantly express RIG-I in their resting state. First we tested the effects of 2-DG on the viability of moDCs. We have found that in contrast to pDCs moDCs tolerate all applied doses of 2-DG (1–50 mM) (Figures 7A,B). For better comparison, to our further experiments we used those 2-DG concentrations (1, 5, and 10 mM) which were also effective in pDCs.

In the next step we observed the maximal expression of mRNA for IFN-α and IFN-β at 12 h in response to RIG-I stimulation (Figures 7C,F), therefore we have studied the effects of glycolysis at this time point. Next we evaluated the impact of 2-DG on the RIG-I-stimulated IFN-α and IFN-β expression at 12 h and found that both the mRNA (Figures 7D,G) and protein levels (Figures 7E,H) are dampened by the inhibition of glycolysis. We also examined the metabolic profile of RIG-I stimulated moDCs in real-time and found a rapid increase in ECAR (Figure 7I) and decrease in OCR (Supplementary Figure 3G) in contrast to pDCs (Figure 4G and Supplementary Figure 1G). Lactate levels and the expression of key glycolytic genes (*LDHA, HK2*, and *HIF1A*) were also elevated following RIG-I stimulation of moDCs suggesting increased glycolytic activity (Figures 7J–M). On the contrary, moDCs were less sensitive to CCCP treatment than pDCs (Supplementary Figures 3A,B). Furthermore, co-treatment of moDCs with specific RIG-I ligand and CCCP, did not alter significantly the type I IFN production as compared to moDCs treated with RIG-I ligand alone (Supplementary Figures 3C–F). Moreover, the RIG-I ligand-exposed moDCs did not display increased mtROS production (Supplementary Figure 3H). All these results indicate that in contrast to pDCs, activation of moDCs via RIG-I results in a metabolic switch from OXPHOS to glycolysis.

### TLR9-Stimulated Primary Human pDCs and RIG-I-Activated moDCs but Not RIG-I-Stimulated pDCs Require Glycolytic Metabolism to Induce Allogeneic Naïve T Cell Proliferation

**Figure 8:**
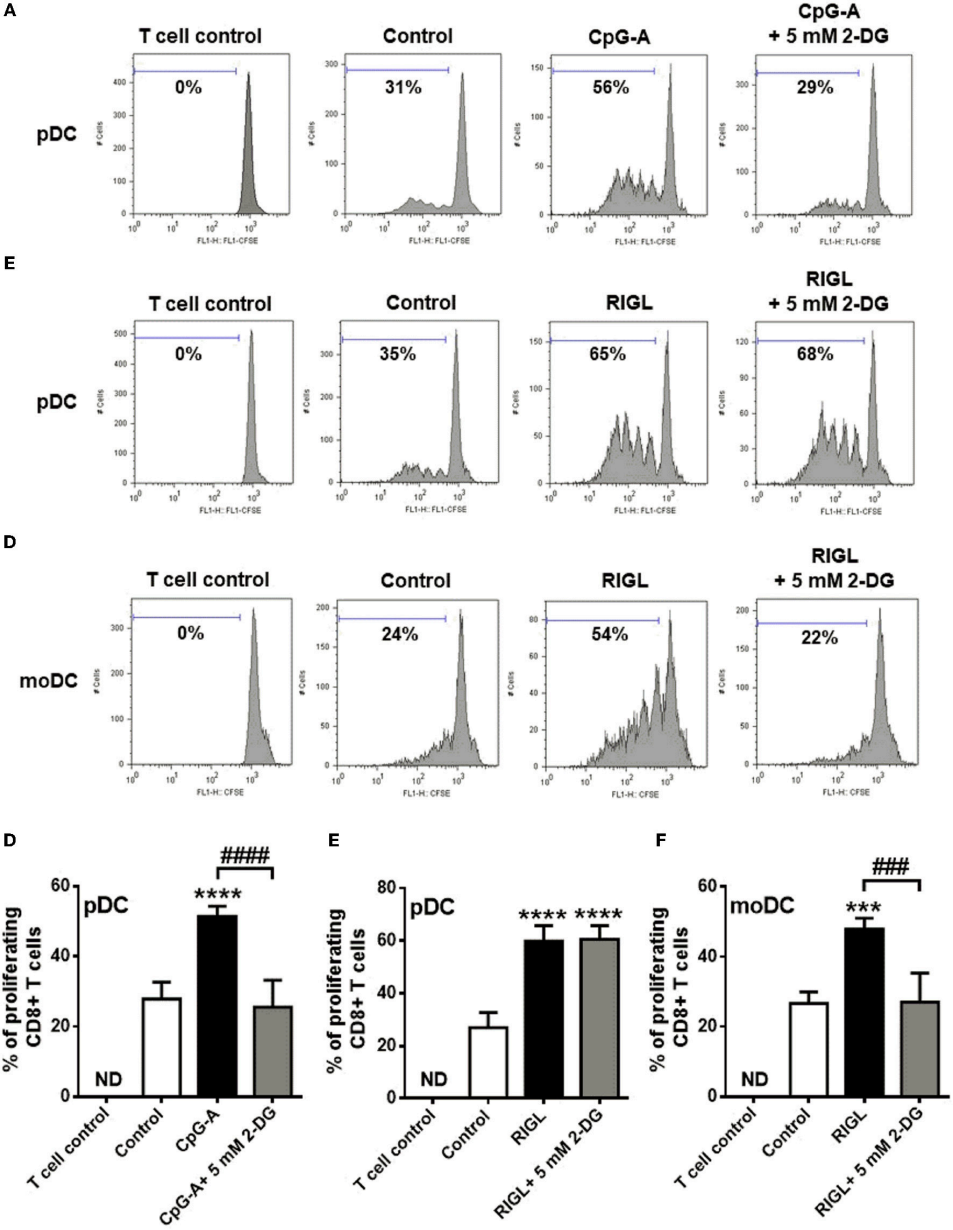
TLR9-stimulated primary human pDCs and RIG-I-activated moDCs but not RIG-I-activated pDC require glycolytic metabolism to induce allogeneic naïve T cell proliferation. **(A–F)** CFSE-labeled allogeneic naïve CD8^+^ T cells were co-cultured with pDCs or moDCs pre-treated with the indicated reagents. After 5 days of co-cultivation, cell division was measured by flow cytometry. **(A–C)** Representative histograms are shown where numbers indicate the percentage of viable dividing CD8^+^ T cells. **(D–F)** Bar graphs represent the mean ± SD of four independent experiments. ***p < 0.001, ****p < 0.0001 vs. control; ^###^*p* < 0.001, ^####^*p* < 0.0001, 2-DG: 2-deoxy-D-glucose; ND, not determined, RIGL, RIG-I ligand.

The transition of DCs from a quiescent into an activated state requires metabolic changes that might also shape their capacity to activate T cells (27). In the present study we have also investigated the impact of metabolism on the capacity of human DCs to interact with T cells. Therefore, highly purified allogeneic naïve CD8^+^ T cells were co-cultured with primary human pDCs as well as moDCs (Figure 8). Prior to co-culturing, pDCs were stimulated with CpG-A or 5′ppp-dsRNA, and moDCs were also exposed to RIG-I ligand in the presence or absence of 5 mM 2-DG or left untreated for 6 h.

Our results show that pDCs treated with TLR9 ligand CpG-A alone induce significant T cell proliferation which process was inhibited when pDCs were activated in the presence of 2-DG (Figures 8A,D). On the contrary, RIG-I-stimulated pDCs induced substantial T cell proliferation which was not influenced by 2-DG treatment of pDCs (Figures 8B,E). However, activation of moDCs with specific RIG-I ligand 5′ppp-dsRNA increased their T cell priming capacity which was significantly impaired upon co-treatment with 2-DG (Figures 8C,F). These observations suggest that the glycolysis in CpG-A-activated pDCs and RIG-I-stimulated moDCs is essential to induce the proliferation of CD8^+^ T cell whereas the T cell priming capacity of RIG-I stimulated pDCs does not depend on it.

## Discussion

DCs are a heterogeneous family of cells that play an essential role in detecting pathogens through a wide array of PRRs such as TLRs and RLRs (28, 29). Ligation of these receptors leads to DC activation characterized by profound changes in gene expression allowing the production of inflammatory mediators as well as the upregulation of costimulatory molecules and major histocompatibility complex (MHC) I and II (30, 31). All these newly acquired properties enable DCs to initiate local inflammation and prime T cell responses (31). A growing body of evidence indicates that stimulation of immune cells including DCs is accompanied by metabolic reprogramming that plays an integral role in their activation process (12, 32). In particular, in response to TLR agonist cDCs and moDCs switch from OXPHOS to glycolysis, the inhibition of which impairs their activation and survival (17, 33, 34). Nevertheless, divergent findings have been reported concerning the role of cellular metabolism in endosomal TLR-mediated pDC activation (18, 20). To our present knowledge, the plausible relation of cellular metabolism to RLR signaling has not been addressed yet, therefore, in this study, we focused on the metabolic profile of RIG-I-stimulated human pDCs.

It has first been described in tumor cells that a metabolic switch from OXPHOS to glycolysis occurs even under normoxic condition to meet energy requirements for cell growth (35). The phenomenon is known as the Warburg effect, which is assumed to be utilized similarly by T-cells to promote proliferation and differentiation into effector cells (36). In contrast, the adoption of Warburg mechanism by innate immune cells, including macrophages and DCs appears to support functional changes such as the secretion of cytokines (12).

The importance of glycolysis in TLR-mediated DC activation has first been recognized by Jantsch et al. (34). They have reported that the TLR4-mediated activation of mouse bone marrow (BM)-derived DCs is highly dependent on glycolysis which is tightly controlled by HIF-1α. Another study demonstrated that stimulation of mouse BM-derived DCs with TLR2, TLR4, and TLR9 ligands promotes aerobe glycolysis that is accompanied by a decrease in mitochondrial activity and OXPHOS (17). Furthermore, it was found that the metabolic switch is supported by the phosphatidylinositol 3-kinase (PI3K)/Akt signaling and inhibited by the adenosine monophosphate-activated protein kinase (AMPK), a regulator of OXPHOS and by the anti-inflammatory cytokine IL-10. In subsequent studies it has been revealed that the early TLR-driven glycolytic reprogramming of BM-derived DCs are mediated via TANK-binding kinase 1 (TBK1), IκB kinase ε (IKKε), and Akt by promoting the association of the glycolytic enzyme HK2 to the mitochondria (27), whereas the long-term commitment to glycolysis is regulated by the mammalian target of rapamycin complex 1 (mTORC1) that induces the expression of HIF1α and inducible nitric oxide synthase (iNOS) (37). Furthermore, the authors propose that the prolonged commitment to glycolysis is only a survival mechanism of iNOS expressing DCs, in which NO production inhibits the mitochondrial electron transport chain (37). Nevertheless, an early increase in glycolytic flux, when iNOS is not active yet, has been suggested to be essential to initiate DC activation in mice. This has been proven by the findings that 2-DG, that inhibits HK2 in the glycolytic pathway, prevented the TLR4-mediated maturation, cytokine, and lactate production of mouse BM-derived DCs at early stages of activation (17, 27). Furthermore, it has been suggested that the rapid increase in glycolysis in TLR-activated DCs might serve the *de novo* synthesis of fatty acids from citrate to support the expansion of organelles required for cytokine/protein synthesis and secretion (27).

Similarly to mouse BM-derived DCs it has been shown that human pDCs also switch to glycolysis to perform antiviral functions (20). The exposure of human pDCs to ssRNA viruses and gardiquimod increased HIF-1α protein expression and induced early glycolysis, whereas decreased OXPHOS activity. Moreover, blockade of glycolysis by 2-DG impaired the TLR7-induced maturation and IFN-α secretion of human pDCs indicating the critical role of glycolysis in pDC antiviral responses. Another study demonstrated that under pathological condition such as imiquimod-induced contact dermatitis, stimulation of both human and murine pDCs with imiquimod resulted in a decrease in OCR, and increase in ECAR, however this was not the case with other TLR7/8 agonist, such as gardiquimod or R848 (19). In this study, we show that activation of human pDCs with the TLR9 agonist CpG-A also leads to an increase in glycolysis as reflected by enhanced ECAR, increased production of lactate and upregulation of glycolytic genes. Moreover, TLR9-induced production of type I IFNs was significantly inhibited by 2-DG highlighting the critical role for glycolysis in the antiviral function of human pDCs. In contrast to our findings, Wu et al. reported that TLR9-driven activation of murine pDCs induced metabolic changes characterized by increased OXPHOS and fatty acid oxidation that was found to be dependent of type I IFNs (18). Furthermore, type I IFN applied alone was also capable to enhance OXPHOS and fatty acid oxidation in murine pDCs (18). Additionally, the authors observed increased basal OCR in pDCs stimulated by imiquimod and in BM-derived DCs activated with the TLR3/MDA5 ligand polyinosinic-polycytidylic acid (polyI:C) (18). On the contrary, *in vivo* stimulation of mouse DCs with polyI:C resulted in metabolic reprogramming toward aerobic glycolysis that has been found to be regulated by type I IFNs (16).

So far, to our knowledge, only one study addressed the connection between cellular metabolism and RLR-mediated signaling (38). The authors used various cell lines (e.g., HEK293, MEF, J774A.1) transfected with plasmids encoding RIG-I to their experiments and described that the RLR-mediated antiviral response requires OXPHOS activity in response to viral infection. In line with this finding, we observed that pDC activation through RIG-I was not accompanied with an increase in glycolysis. Furthermore, RIG-I-mediated production of type I IFN was increased by 2-DG, whereas reduced by CCCP, a chemical inhibitor of OXPHOS in human pDCs. Our observations are consistent with the report of Yoshizumi et al. showing that arresting OXPHOS activity by CCCP disrupts RLR-mediated signaling in HEK293 cells (38). We also observed a minimal increase in OCR upon RIG-I stimulation that further support the idea that RIG-I-stimulated human pDCs rely on OXPHOS to fulfill their function.

It is worth to mention that the low dose of CpG-A applied to induce RIG-I in pDCs does not induce type I IFN production, however can promote a shift toward glycolysis (data not shown). Nevertheless, we suppose based on our results that this glycolytic shift might be a transient change and pDCs can increase their OXPHOS activity upon RIG-I stimulation.

Studies on human moDCs revealed that immature and tolerogenic moDCs display metabolic signatures of OXPHOS, fatty acid oxidation and glycolysis, whereas mature moDCs show higher glycolytic rate mirrored by increased lactate production (33). In contrast to murine mature BM-derived DCs, where the switch leads to a total blockade of OXPHOS and thus shows a complete dependence on glycolysis for energy production and survival (37), mature human moDCs still display a limited OXPHOS activity that is able to provide energy (33). In line with our observations the authors found that 50 mM 2-DG resulted only in a slight decrease in cell viability indicating high metabolic adaptation for survival. Interestingly, mature and immature moDCs showed similar levels of iNOS expression and NO production suggesting that the TLR-induced decrease in mitochondrial activity is NO-independent in human moDCs (33) in contrast to mouse DCs (37). We found that, in contrast to pDCs, human moDCs stimulated via RIG-I increased lactate release, upregulated the expression of glycolytic related genes, and displayed higher ECAR and reduced OCR. While CCCP treatment did not have any significant effects, blockade of glycolysis by 2-DG impaired significantly the type I IFN production of moDC indicating a dependence on glycolytic metabolism rather than OXPHOS.

In addition, we observed remarkable differences between primary pDCs and moDCs concerning their mtROS production as RIG-I stimulation increased mtROS levels only in pDCs but not in moDCs. Interestingly, blockade of glycolysis increased the RIG-I-triggered type I IFN secretion in pDCs, whereas decreased it in moDCs. These results imply that, in contrast to moDCs, the defect of glycolysis in pDCs promotes OXPHOS activity that can result in increased mtROS production. Previously we have described that elevated levels of mtROS support the RIG-I-mediated responses in pDCs (25), thus we hypothesize that this might be the reason behind the increased type I IFN production of pDCs co-treated with RIG-I ligand and glycolysis inhibitor.

So far, limited data are available concerning the impact of metabolism on the capacity of human DCs to interact with T cells. It has been reported that *in vivo* activation of murine DCs in the presence of 2-DG impaired their CD4^+^ and CD8^+^ T cell stimulatory capacity demonstrating a crucial role for TLR-induced glycolysis in the priming functions of DCs (27).

Our data also suggest an essential role for glycolytic metabolism in the priming function of TLR9-activated human pDCs and RIG-I-stimulated moDCs. Interestingly, treatment of pDCs with 2-DG did not have any effect on the allogeneic CD8+ T cell priming capacity of RIG-I-stimulated pDCs. All these data suggest that the immunogenic capacity of different DC subtypes coincides with their divergent metabolic demands.

In conclusion we show that different DC subtypes such as human pDCs and moDCs have distinct metabolic requirements. In response to RIG-I stimulation moDCs switch to glycolysis whereas pDCs seems to rely on OXPHOS rather than glycolysis. These differences might be explained by the fact that these two DC subtypes possess different viral sensor repertoire which elicit divergent antiviral responses. Plasmacytoid DCs apply endosomal TLRs in the early phases of virus infection and use RIG-I only in the later stages of antiviral responses. On the contrary, moDCs engage both TLRs and RLRs during the initial viral encounter which, as we suppose, requires a switch to glycolysis to expand endoplasmic reticulum and Golgi for the large-scale production of antiviral proteins (27). Furthermore, our data imply that cellular metabolism controls the T cell priming function of human DCs indicating that metabolic manipulation of DCs might be used to modulate their immune-polarizing properties as well. Overall, altering human DC functionality through metabolic modulation requires a more comprehensive knowledge and understanding due to the complexity and diversity of antiviral responses induced by various PRRs.

## Author Contributions

KP and TF designed the research, performed experiments, analyzed and interpreted data, and wrote the manuscript. MS, DB, AM, and AS performed experiments and participated in data analysis. KP, AB, and TB contributed with essential reagents. All authors reviewed and approved the manuscript.

### Conflict of Interest Statement

The authors declare that the research was conducted in the absence of any commercial or financial relationships that could be construed as a potential conflict of interest.

## Abbreviations

2-DG: 2-deoxy-D-glucose
CCCP: carbonyl cyanide m-chlorophenyl hydrazone
**cDC**: conventional DC
DC: dendritic cell
ECAR: extracellular acidification rate
HIF1A: hypoxia-inducible factor 1-alpha
**HK2**: hexokinase 2
IFN: interferon
LDHA: lactate dehydrogenase A
moDC: monocyte-derived DC
mtROS: mitochondrial reactive oxygen species
OCR: oxygen consumption rate
OXPHOS: oxidative phosphorylation
pDC: plasmacytoid DC
RIG-I: retinoic-acid inducible gene I
RLR: RIG-I-like receptors
TLR: Toll-like receptors.
