# Supplementary material for: Human Plasmacytoid and Monocyte-Derived Dendritic Cells Display Distinct Metabolic Profile Upon RIG-I Activation
Source: Front Immunol. 2018 Dec 21;9:3070. doi: 10.3389/fimmu.2018.03070 (PMC6308321; doi:10.3389/fimmu.2018.03070)
Supplement: Supplementary file 1 [file Data_Sheet_1.pdf]

Supplementary Material

Human plasmacytoid and monocyte-derived dendritic cells display distinct metabolic profile upon RIG-I activation

Tünde Fekete, Mate Sütő, Dora Bencze, Anett Mázló, Attila Szabo, Tamas Biro, Attila Bacsi, Kitti Pazmandi*

* **Correspondence:** Kitti Pazmandi: pazmandikitti@yahoo.de

Supplementary Figures

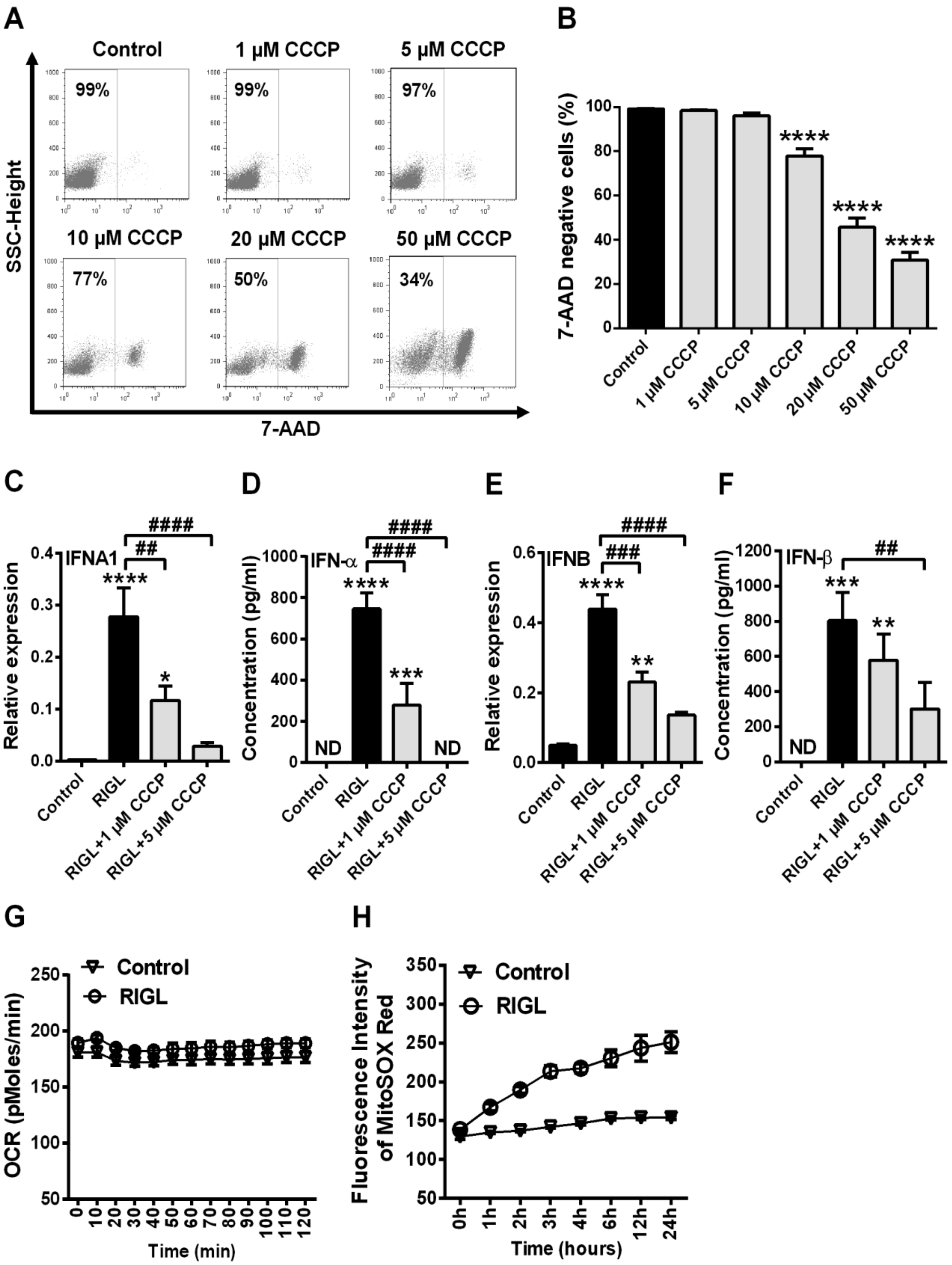

A
Control
1 μM CCCP
5 μM CCCP
10 μM CCCP
20 μM CCCP
50 μM CCCP
99%
99%
97%
77%
50%
34%
SSC-Height
7-AAD
B
7-AAD negative cells (%)
****
Control
1 μM CCCP
5 μM CCCP
10 μM CCCP
20 μM CCCP
50 μM CCCP
C
IFNA1
Relative expression
####
##
****
*
Control
RIGL
RIGL+1 μM CCCP
RIGL+5 μM CCCP
D
IFN-α
Concentration (pg/ml)
ND
***
E
IFNB
###
**
F
IFN-β
G
OCR (pMoles/min)
Time (min)
Control
RIGL
H
Fluorescence Intensity of MitoSOX Red
Time (hours)

Supplementary Figure 1. Inhibition of oxidative phosphorylation (OXPHOS) affects the RIG-I-mediated type I IFN production in GEN2.2 cells. (**A**,**B**) GEN2.2 cells were treated with increasing concentration of mitochondrial uncoupler carbonylcyanide m-chlorophenylhydrazone (CCCP; 1-50 μM), then cell viability was analyzed by flow cytometry. (**C**-**F**) GEN2.2 cells were pre-treated with 0.25 μM of CpG-A for 16 hours then following thorough washing steps stimulated with the RIG-I agonist 5'ppp-dsRNA (RIGL, 1 μg/ml) in the presence or absence of CCCP (1 and 5 μM). The expression of IFN-α and IFN-β was measured at the mRNA level by Q-PCR at 3 hours (**C**,**E**) and at the protein level by ELISA at 6 hours (**D**,**F**). (**G**) Following activation with 5'ppp-dsRNA real-time OCR was determined by EFA. The results of a representative experiment are shown. (**H**) The mtROS production was detected by MitoSOX^{TM} Red mitochondrial superoxide indicator. The results of a representative experiment are shown. (**A**) Representative dot plots are shown and numbers indicate the percentage of 7-aminoactinomycin D (7-AAD) negative cells. (**B**-**F**) Data represent the mean ± SD of at least 3 individual experiments. *$p<0.05$, **$p<0.01$, ***$p<0.001$, ****$p<0.0001$ vs. control; $^{\#\#}p<0.01$, $^{\#\#\#}p<0.001$, $^{\#\#\#\#}p<0.0001$, ND: not determined

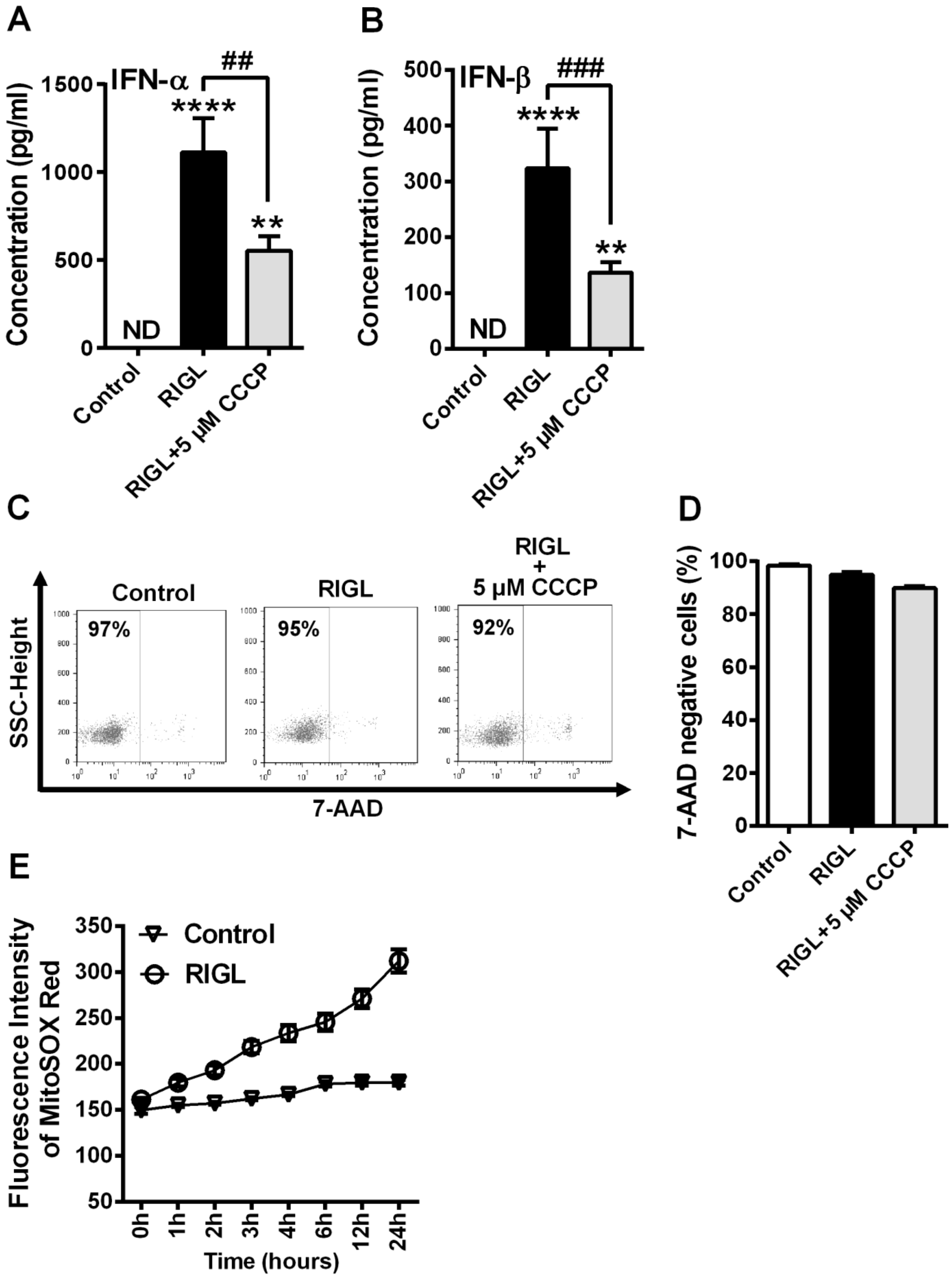
A
IFN-α
##
****
**
Concentration (pg/ml)
1500
1000
500
0
ND
Control
RIGL
RIGL+5 μM CCCP
B
IFN-β
###
****
**
Concentration (pg/ml)
500
400
300
200
100
0
ND
Control
RIGL
RIGL+5 μM CCCP
C
Control
RIGL
RIGL + 5 μM CCCP
97%
95%
92%
SSC-Height
7-AAD
D
7-AAD negative cells (%)
100
80
60
40
20
0
Control
RIGL
RIGL+5 μM CCCP
E
Control
RIGL
Fluorescence Intensity of MitoSOX Red
350
300
250
200
150
100
50
0h
1h
2h
3h
4h
6h
12h
24h
Time (hours)

Supplementary Figure 2. Inhibition of oxidative phosphorylation (OXPHOS) affects the RIG-I-mediated type I IFN production in primary human pDCs. Freshly isolated primary human pDCs were pre-treated with CpG-A for 16 hours then following thorough washing steps stimulated with the RIG-I agonist 5’ppp-dsRNA in the presence or absence of 5 μM CCCP. (**A**,**B**) IFN-α and IFN-β protein levels were measured from the supernatants at 6 hours. (**C**,**D**) Cell viability was measured by 7-aminoactinomycin D (7-AAD) staining using flow cytometry. (**E**) The mtROS production was detected by MitoSOX™ Red mitochondrial superoxide indicator. The results of a representative experiment are shown. (**C**) Representative dot plots are shown and numbers indicate the percentage of 7-AAD negative cells. (**A**,**B**,**D**) Data represent the mean ± SD of at least 3 individual experiments. $^{**}p<0.01$, $^{****}p<0.0001$ vs. control; $^{\#\#}p<0.01$, $^{\#\#\#}p<0.001$, ND: not determined

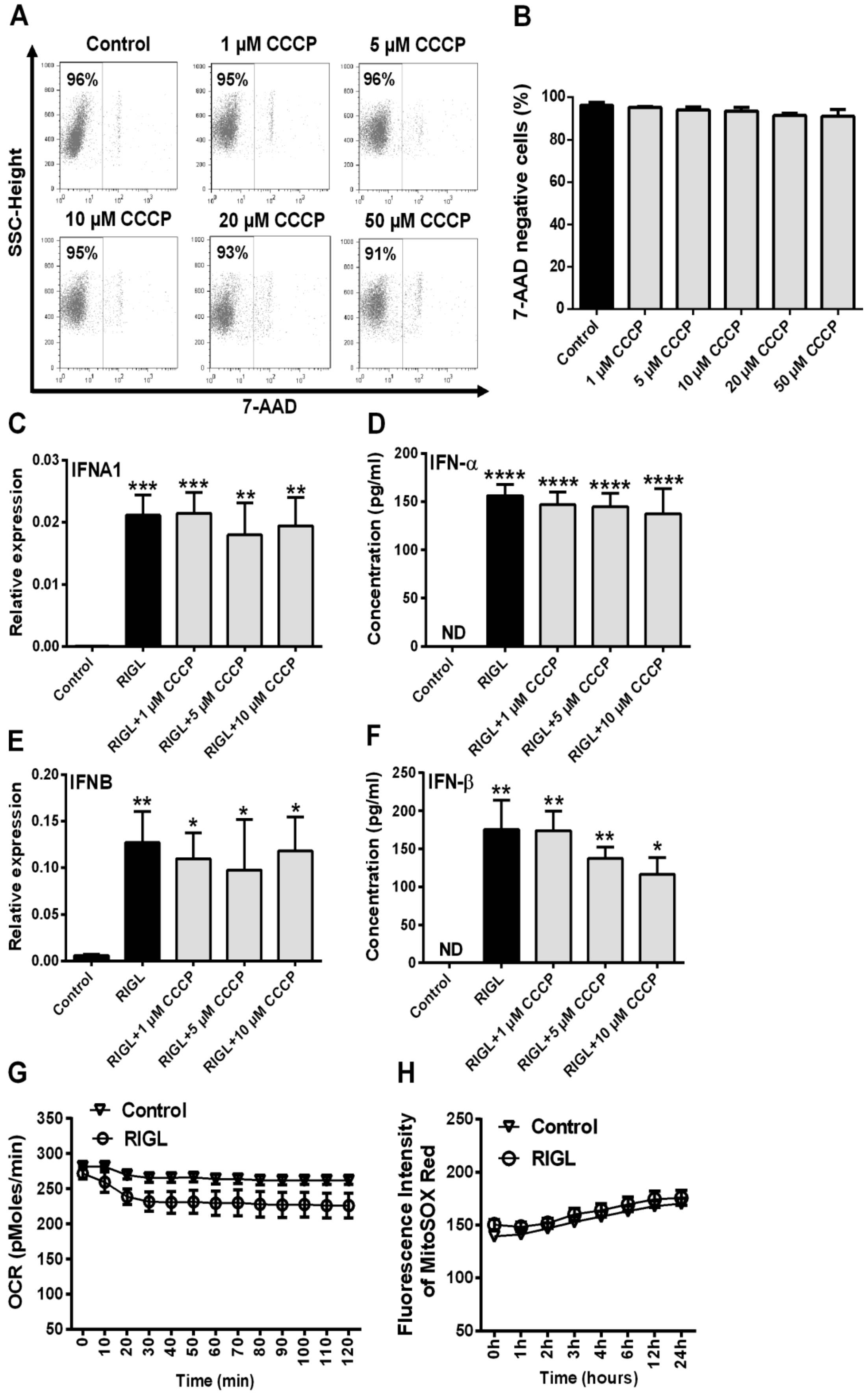
A
Control
1 μM CCCP
5 μM CCCP
10 μM CCCP
20 μM CCCP
50 μM CCCP
96%
95%
96%
95%
93%
91%
SSC-Height
7-AAD
B
7-AAD negative cells (%)
Control
1 μM CCCP
5 μM CCCP
10 μM CCCP
20 μM CCCP
50 μM CCCP
C
IFNA1
Relative expression
Control
RIGL
RIGL+1 μM CCCP
RIGL+5 μM CCCP
RIGL+10 μM CCCP
D
IFN-α
Concentration (pg/ml)
ND
E
IFNB
Relative expression
F
IFN-β
Concentration (pg/ml)
ND
G
Control
RIGL
OCR (pMoles/min)
Time (min)
H
Control
RIGL
Fluorescence Intensity of MitoSOX Red
Time (hours)

Supplementary Figure 3. Inhibition of oxidative phosphorylation (OXPHOS) does not affect the viability and RIG-I-mediated type I IFN production in human immature moDCs. (**A**,**B**) moDCs were treated with increasing concentration of the mitochondrial uncoupler carbonylcyanide m-chlorophenylhydrazone (CCCP; 1-50 μM) then cell viability was analyzed by flow cytometry. (**C**-**F**) Cells were left untreated or stimulated with the RIG-I agonist 5'ppp-dsRNA (RIGL, 1 μg/ml) in the presence or absence of CCCP (1, 5 and 10 μM). After 12 hours the expression of IFN-α and IFN-β was measured at the mRNA level by Q-PCR (**C**,**E**) and at the protein level by ELISA (**D**,**F**). (**G**) Following activation with 5'ppp-dsRNA real-time OCR was determined by EFA. The results of a representative experiment are shown. (**H**) The mtROS production was detected using MitoSOX^{TM} Red mitochondrial superoxide indicator. The results of a representative experiment are shown. (**A**) Representative dot plots are shown and numbers indicate the percentage of 7-aminoactinomycin D (7-AAD) negative cells. (**B**-**F**) Data represent the mean ± SD of at least 3 individual experiments. *p<0.05, **p<0.01, ***p<0.001, ****p<0.0001 vs. control, ND: not determined
